# State- and Trait-Related Alterations of Motor Cortex Excitability in Tinnitus Patients

**DOI:** 10.1371/journal.pone.0085015

**Published:** 2014-01-07

**Authors:** Martin Schecklmann, Michael Landgrebe, Tobias Kleinjung, Elmar Frank, Rainer Rupprecht, Philipp G. Sand, Peter Eichhammer, Göran Hajak, Berthold Langguth

**Affiliations:** 1 Department of Psychiatry and Psychotherapy, University Regensburg, Regensburg, Germany; 2 Department of Psychiatry, Psychosomatic Medicine and Psychotherapy, Social Foundation Bamberg, Bamberg, Germany; 3 Department of Otorhinolaryngology, University of Zurich, Zurich, Switzerland; University of Wuerzburg, Germany

## Abstract

Chronic tinnitus is a brain network disorder with involvement of auditory and non-auditory areas. Repetitive transcranial magnetic stimulation (rTMS) over the temporal cortex has been investigated for the treatment of tinnitus. Several small studies suggest that motor cortex excitability is altered in people with tinnitus. We retrospectively analysed data from 231 patients with chronic tinnitus and 120 healthy controls by pooling data from different studies. Variables of interest were resting motor threshold (RMT), short-interval intra-cortical inhibition (SICI), intra-cortical facilitation (ICF), and cortical silent period (CSP). 118 patients were tested twice - before and after ten rTMS treatment sessions over the left temporal cortex. In tinnitus patients SICI and ICF were increased and CSP was shortened as compared to healthy controls. There was no group difference in RMT. Treatment related amelioration of tinnitus symptoms were correlated with normalisations in SICI. These findings confirm earlier studies of abnormal motor cortex excitability in tinnitus patients. Moreover our longitudinal data suggest that altered SICI may reflect a state parameter, whereas CSP and ICF may rather mirror a trait-like predisposing factor of tinnitus. These findings are new and innovative as they enlarge the knowledge about basic physiologic and neuroplastic processes in tinnitus.

## Introduction

Tinnitus is associated with neural changes in both the auditory pathway (increase of spontaneous stochastic firing rate, hyperactivity, alterations of the tonotopic map), and in non-auditory brain areas [1]. Tinnitus related changes of brain activity and connectivity (e.g. [2], [3]) seem to reflect pathologically altered brain networks, which develops as a response to abnormal sensory input [4]. Based on these findings repetitive transcranial magnetic stimulation (rTMS) was introduced as a treatment approach in tinnitus [5].

In addition TMS can also be used as a diagnostic tool for the assessment of motor cortex excitability by quantifying contractions of peripheral muscles induced by stimulation of the corresponding motor cortex representation. Assessment of motor cortex excitability in a longitudinal study design before and after a specific therapeutic intervention (e.g. multiple sessions of rTMS) enables to describe treatment-related neuroplastic changes.

Several pilot studies suggest that tinnitus is related to alterations in motor cortex excitability. ICF was found to be enhanced in 19 patients with tinnitus in contrast to 19 controls [6] and CSP was found to be shortened (37 patients vs. 12 controls) [7]. However, it is not known whether these findings are state or trait parameters. They could either represent cross-modal plasticity like changes accompanying tinnitus, or unspecific general neural mechanisms reflecting individual predisposition factors for developing tinnitus.

Moreover it has been shown, that rTMS over the temporal cortex can induce changes in motor cortex excitability. Five days of active low-frequency rTMS over auditory cortex lead to prolongation of the CSP in 18 healthy subjects [8], whereas sham rTMS had not such effects. Clinical benefit of the same protocol in ten patients with tinnitus was positively correlated with changes in SICI, ICF, and CSP [9].

Here, we aimed to investigate in a retrospective analysis whether the observed alterations of cortical excitability can be replicated in a large sample of patients with tinnitus and controls. In order to address the open question whether such changes represent state or trait markers, we analysed longitudinal data before and after a therapeutic intervention.

## Materials and Methods

### Ethics Statement

All participants gave written informed consent after a comprehensive explanation of the procedures. All studies whose data contributed to this analysis were approved by the Ethics Committee at the University of Regensburg. All experiments were conducted in accordance with the last revision of the Declaration of Helsinki.

### Subjects

Patients presenting with a phantom sound of at least two month duration were recruited and investigated at the Tinnitus center Regensburg (Germany) which is a collaboration of the Department of Otolaryngology and the Department of Psychiatry and Psychotherapy of the University Hospital of Regensburg (Germany). Measurements of cortical excitability were performed in 231 patients (172 (74.5%) males; 49.5±12.2 (20–83) years) with chronic tinnitus (duration 93±95 (2–476) months). 44 (19%) out of 208 patients reported a purely left-sided, 37 (16%) a purely right-sided tinnitus, and 127 (55%) patients described their tinnitus as bilateral or originating within the head. Tinnitus distress was assessed by the German version [10] of the Tinnitus questionnaire (TQ) [11]. TQ overall scores ranged from 3 to 79 (41±18). Patients suffering from Menière’s disease, presenting conductive hearing loss or displaying hints of objective tinnitus were not included. 132 patients underwent a complete otologic and audiologic examination including pure tone audiometry, tympanometry, stapedius reflex tests, and otomicroscopy. For the other subjects no standardized audiometry data were available. Mean hearing level in the frequency range from 125 to 8000 Hz was 17±12 dB HL (0–61). As patients were measured in the context of clinical trials of rTMS only patients were included that were eligible for rTMS treatment. Thus, patients with cardiac pacemakers, history of seizures, suspected diagnosis of organic brain damage or any other severe somatic, neurologic, or psychiatric diagnosis were not included.

Healthy volunteers (n = 120; 57 (47.5%) males; age: 28.2±7.5 (19–55) years) were recruited through advertisements and by requesting the hospital staff. All healthy participants were free of medication and free of any mental disorder as verified during an interview by board-certified psychiatrists (B.L., E.F. and P.E.). Controls did significantly differ from patients with respect to age (t = 17.5; df = 349; p<0.001) and gender (η^2^ = 25.3; df = 1; p<0.001).

### Procedures

For measurement of cortical excitability, the participants were seated in a reclining chair. TMS was delivered by two Magstim 200 stimulators (Magstim Co., UK) connected via a Bistim module to a figure-of-eight coil (double-circular-70-mm coil). The coil was held tangential to the skull and with the handle pointing backwards and about 45° away from the midline. The optimal coil position for stimulation was defined as the position above the left motor cortex for eliciting MEPs of maximal amplitude in abductor digiti minimi muscle with a slightly supra-threshold stimulus. Once this position was found, it was marked on a scientific head cap and the coil was held in this position by the investigator.

MEPs of the abductor digiti minimi of the right hand were recorded with surface electrodes. The analogue signal was registered, band-pass filtered between 20 Hz and 10 kHz, then digitised at a frequency of 5 kHz and analysed off-line. The employed hardware setup required the analogue signal to be acquired with a band-pass filter between 20 Hz and 10 kHz and a digitization frequency of 5 kHz. Whereas these suboptimal filter settings yield the possibility of aliasing effects (according to the Nyquist theorem, a 2.5 kHz low pass filter should have been applied to the analogue signal), the small signal amplitudes for high frequencies render it unlikely that the recording setup may have biased or altered the present findings [12].

Resting and active motor threshold were determined according to Rossini and colleagues [13]. Resting motor threshold (RMT) was determined as the lowest stimulation intensity that evoked in at least four out of eight consecutive trials a MEP of at least 50 µV in the resting abductor digiti minimi. Active motor threshold was determined only for conduction of the double-pulse rTMS paradigms (ICI and ICF) and was not included in analysis due to high variability. Active motor threshold was defined as the lowest stimulation intensity that evoked in at least four out of eight consecutive trials a MEP of at least 250 µV during isometric contraction of the abductor digiti minimi at about 20% of maximum voluntary contraction. A constant level of voluntary contraction was maintained by audiovisual feedback of the electromyographic (EMG) activity. MEP amplitudes were measured peak-to-peak.

Short-interval intra-cortical inhibition and intracortical facilitation were measured with a paired-pulse TMS protocol [14]. The intensity of the first (conditioning) stimulus was set at 90% of the active motor threshold. The second (test) stimulus was delivered at an intensity that produced MEPs of about 1 mV in the resting abductor digiti minimi. Interstimulus intervals (ISIs) were 2 ms and 15 ms to measure short-interval intra-cortical inhibition (reduction of amplitude) and intracortical facilitation (increase of amplitude), respectively [14]. The conditioned stimuli and the control condition (test pulse alone) were each tested 10 times in a random order (inter-sweep-interval: 4 s). The effect of conditioning stimuli on MEP amplitude at each ISI was determined as the ratio of the average amplitude of conditioned MEP to the average amplitude of unconditioned test MEP.

Cortical silent period was measured in 10 trials (stimulus intensity: 150% resting motor threshold; inter-sweep-interval: 5 s) in the moderately active abductor digiti minimi muscle on the non-rectified recording of every individual sweep and then averaged [15]. Participants were instructed to contract this muscle at 30% maximum strength. A constant level of voluntary contraction was maintained by audiovisual feedback of the electromyographic (EMG) activity. The onset of the cortical silent period was defined as the end of the MEP when activity dropped consistently below pre-stimulus EMG level. The end of the cortical silent period was defined as first reappearance of voluntary EMG activity. In conclusion, TMS variables of interest were resting motor threshold (RMT), cMEP/uMEP ratio of 2 ms and 15 ms interstimulus intervals (SICI and ICF, respectively), and cortical silent period (CSP).

### Study Procedures

The therapeutic intervention consisted of 10 rTMS sessions on consecutive weekdays. Treatment effects were evaluated by change in TQ between the first and the last day of treatment. Motor cortex excitability was examined on these days, too. We analyzed longitudinal data of 118 patients participating in three different treatments [16], [17] and receiving three different active stimulation protocols (1000 stimuli over auditory cortex with 1 Hz: n = 76; 4000 stimuli over auditory cortex with 1 Hz: n = 26; 2000 stimuli over left frontal cortex with 20 Hz and 2000 stimuli over auditory cortex with 1 Hz: n = 23). 1 Hz stimulation was performed over the left auditory cortex. In single cases of positron emission guided stimulation the right auditory cortex was stimulated instead. Stimulation intensity was set to 110% RMT.

### Statistics

Firstly, we were interested in differences in motor cortex excitability between patients with tinnitus and healthy controls. We contrasted both groups with two-sided Student t-tests. As groups were not comparable for age and gender, we also contrasted both groups with an analysis of variance (ANOVA) with group as between-subjects factor and age and gender as covariate (ANCOVA). As we included data from former studies [6], [8], [9] we controlled for this fact by including a dummy coded variable as third covariate. Data of 28 (11.1%) patients and 17 (14.2%) controls overlapped with former studies. To evaluate the role of hearing loss, we correlated mean hearing level for the left and right ear with measures of motor cortex excitability in a sub-group of 132 patients.

Secondly, we were interested if rTMS induced clinical amelioration was associated with changes in motor cortex excitability. Thus, we correlated changes/differences in the TQ with changes/differences in excitability parameters. This analysis would shed light on the question if tinnitus-related changes in motor cortex excitability represent state or trait markers of tinnitus. As three different treatments were included we were interested in the interrelationship of kind of treatment with correlations. Thus, we repeated the correlation analysis with partial correlations using kind of treatment as control variable.

As we had four variables of interest (RMT, SICI, ICF, and CSP), only p-values below a Bonferroni corrected significance level of 0.0125 (0.05/4 = 0.0125) were presented as significant. Statistical analyses were performed with SPSS 18.0.0 (SPSS, USA).

## Results

Firstly, group contrasts of two big samples of patients with tinnitus (n = 231) and healthy controls (n = 120) revealed significant differences in short-interval intra-cortical inhibition (SICI), intra-cortical facilitation (ICF; with a statistical trend), and cortical silent period (CSP). Patients with tinnitus showed increased SICI (by definition, lower values of SICI correspond to enhanced inhibitory function), increased intra-cortical facilitation, and shortened cortical silent period (table 1). As groups differed with respect to age and gender and we included data from previous publications, we controlled for these potential confounders by ANCOVAs. Effects were still significant. We did not find significant effects for resting motor threshold (RMT). Correlations between hearing level and RMT (right: r = 0.015; p = 0.866; left: r = 0.076; p = 0.389), SICI (right: r = 0.136; p = 0.120; left: r = 0.103; p = 0.239), ICF (right: r = −0.139; p = 0.112; left: r = −0.116; p = 0.186), and CSP (right: r = −0.001; p = 0.993; left: r = 0.034; p = 0.701) were not significant.

**Table 1:** Descriptive and statistical data of motor cortex excitability for the group contrasts.

	tinnitus	controls	t-test	ANCOVA#
resting motor threshold	43.21±7.84	43.50±7.53	t = 0.328; df = 346; p = 0.743; d = 0.037	t-test not significant
short-interval intra-cortical inhibition	0.47±0.37	0.58±0.30	t = 2.801; df = 349; p = 0.005; d = 0.316*	F = 3.202; df = 1,346; p = 0.009*
intra-cortical facilitation	1.47±0.78	1.29±0.37	t = 2.385; df = 349; p = 0.018; d = 0.269+	F = 3.400; df = 1,346; p = 0.010*
cortical silent period (s)	0.11±0.05	0.13±0.04	t = 4.612; df = 335; p<0.001; d = 0.541*	F = 2.843; df = 1,332; p = 0.008*

significant at a Bonferroni corrected threshold with p-values displayed uncorrected;

near significant at a Bonferroni corrected threshold with p-values displayed uncorrected;

^#^ indicates group effects of an ANCOVA (analysis of variance) with age, gender, and former analysis as covariate.

**Figure 1:**
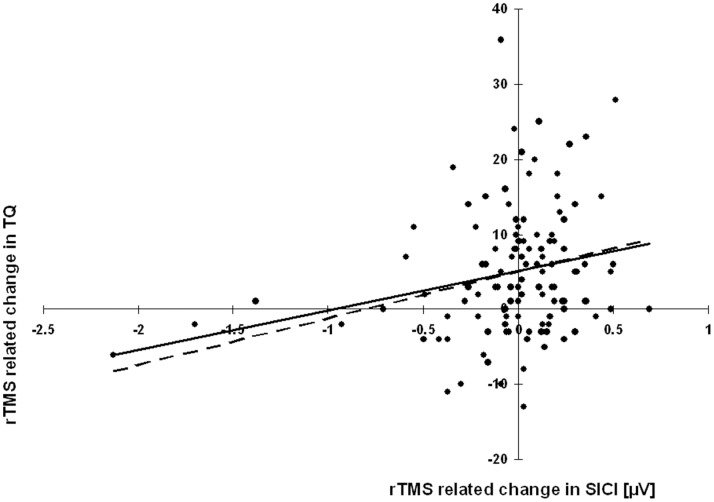
Scatter plot for the significant Pearson correlation between the repetitive transcranial magnetic stimulation (rTMS) induced change in short-interval intra-cortical inhibition (SICI) and rTMS induced change in tinnitus questionnaire (TQ) including the regression line for the whole sample (continuous line) and for the sample without the three outliers (dashed line).

Secondly, for a sub-group of patients (n = 118) data of motor cortex excitability were available at two different time points before and after a therapeutic intervention. Here we found that a change in tinnitus severity correlated significantly with changes in SICI, i.e., the higher the improvement the higher the decrease in SICI (r = 0.234; n = 116; p = 0.011). A scatter plot (figure 1) indicated three outliers with high SICIs (negative values of the abscissa). Excluding these patients from the analysis did also reveal a significant correlation (r = 0.188; n = 113; p = 0.046). There were no significant correlations of RMT (r = 0.062; n = 116; p = 0.509), ICF (r = 0.124; n = 116; p = 0.183), or CSP (r = 0.043; n = 115; p = 0.648). Partial correlations with kind of treatment as covariate indicates no systematic differences in results (RMT: r = 0.056; n = 112; p = 0.551; SICI: r = 0.237; n = 111; p = 0.011; ICF: r = 0.121; n = 111; p = 0.200; CSP: r = 0.04; n = 112; p = 0.640) which means that correlations were comparable for all three kind of treatments.

## Discussion

Motor cortex excitability as measured by short-interval intra-cortical inhibition (SICI), intra-cortical facilitation (ICF), and cortical silent period (CSP) appeared to be altered in tinnitus patients (table 1). SICI and ICF were increased, and CSP was shortened. These findings confirm findings from previous studies that demonstrated increased ICF [6] and shortened CSP [7] in smaller samples of tinnitus patients.

SICI and CSP are measures for inhibitory and ICF of facilitatory processes in the motor system. Tinnitus is known to be related to neural changes along the auditory pathway emerging from a dysbalance of inhibitory and excitatory neurons [18]. Activity in the central auditory pathways is further modulated by inter-connected extra-auditory areas such as frontal regions as parts of “noise-cancellation” or top-down inhibition processes [3], [19], [20]. However in current pathophysiological models of tinnitus the motor system is not considered to be directly involved [4]. Notably the findings of abnormal motor cortex excitability [6], [7] do not prove specific involvement of the motor cortex in tinnitus pathophysiology, since these findings may reflect global changes. On the other hand there are several hints for a possible involvement of the motor cortex in tinnitus. In primates the relevance of the motor system for auditory perception has been demonstrated repeatedly [21], [22]. Moreover, electroencephalographic studies revealed alterations in oscillatory activity in the supplementary motor area related to different aspects of tinnitus [23].

Several confounding factors may potentially influence motor cortex excitability in tinnitus patients. First, the influence of hearing loss has to be considered as a confounding factor. Hearing loss results in sensory deprivation and is considered to be the most relevant risk factor for tinnitus. At the same time sensory deprivation may have a direct influence on motor cortex excitability. Increased ICF has been reported after experimental somatosensory deafferentiation [24] and in limb amputees [25], [26]. Sensory deprivation in the auditory system could induce changes in the motor system via mechanisms of cross-modal plasticity (see below). In order to control for hearing loss we analysed the subsample for which audiometric data were available (132 patients; 57% of the whole sample). However, we did not find significant correlations between hearing level and motor cortex excitability. Other influencing factors might be co-morbid pain syndromes and the ability to influence the tinnitus percept by somatic manoeuvres [27]. This is more frequently the case in tinnitus patients with temporomandibular joint complaints [28], [29]. Whether the ability to modulate tinnitus by somatic manoeuvres has an impact on motor cortex excitability could not be analysed in the present study, since these data were not available. Further limitations of this analysis are that we did not control for conditions which are known to interfere with motor cortex excitability and plasticity or with tinnitus or both. For example, mental diseases such as anxiety or affective disorders are known in tinnitus as co-morbid condition [30] but also in modulating motor cortex excitability [12]. The same is the case for medication (e.g., GABAergic drugs) which can interfere with TMS related measures [31] and tinnitus [32]. Insomnia [33] and also hyperacousis [34] also are closely related to tinnitus. With respect to insomnia it could be demonstrated that selective REM and complete night sleep deprivation is accompanied by changes in SICI [35], [36]. Thus, further studies might control for this limitation by asking for hours of sleep per night. More generally, level of education/intelligence, sex [37], age [38], and TMS procedures such as differences in maximum voluntary contraction for AMT, stimulation intensity and ISI for SICI, no use of neuronavigation might also have influence on the presented findings. We statistically controlled the factors age and sex by redoing the group contrasts with ANCOVAs. Methodologically, future studies might try to control for these demographic factors by matching the groups with respect to these variables. TMS parameters generally should be reported in detail [39]. Thus, future studies should carefully control for these conditions.

Alterations of motor cortex excitability in a cross-sectional group comparison between tinnitus patients and controls could be either a consequence or a pre-condition of tinnitus or both. This could be evaluated by large-sampled prospective studies in non-tinnitus sufferers. Another approach for disentangling state and trait markers is to evaluate the influence of treatment effects. Here the cross-sectional comparison between tinnitus patients and controls was complemented by a longitudinal data analysis in order to identify to which extent the observed alterations of motor cortex excitability in tinnitus patients reflect an alteration of inhibitory-excitatory cortical processes predisposing for tinnitus, or a consequence of tinnitus.

By analysing longitudinal data before and after rTMS treatment we found a significant association of treatment-related change in TQ with change in SICI, i.e., the higher amelioration of symptoms the higher the decrease in inhibition (figure 1). Pending confirmation by further studies our findings suggest that the increase of SICI might display a state-like factor for tinnitus severity, whereas increased ICF and shortened CSP might display rather trait-like factors. Speculatively, increased ICF and shortened CSP may reflect neural hyper-excitability as a general risk factor for developing a hyper-excitability disorder such as tinnitus. The increased SICI in contrast might reflect a compensatory effort related to the severity of tinnitus.

Alternatively, the increased SICI may reflect a direct consequence of tinnitus. Neural changes in the auditory system could induce changes in the motor system via mechanisms of cross-modal plasticity. Functional imaging and transcranial magnetic stimulation studies suggest multisensory association areas as neural basis for cross-modal or multisensory integration. However, recent findings in synaesthesia - an example for the incorporation of one modality in two modalities - changed the focus of research from a single core region to network properties and connectivity [40]. Also primary visual and auditory areas were identified as synaesthesia relevant [41]. Another example for the cross-modal functioning of the brain is transfer of processes from a sensory system to a deprived sensory system, e.g., deaf people show active auditory cortex during visual stimulation [42], [43]. Considered mechanisms may take place via multi-sensory association areas, via direct cortico-cortical connections, or via subcortical interplay of the corresponding sensory systems at the thalamic level [42]. In tinnitus and other neuropsychiatric disorders, the model of thalamo-cortical dysrhythmia considers altered thalamic activity due to faulty signals in afferent neurons as the core symptom. This altered thalamic activity in turn is generating altered cortical activity which again induces symptoms such as phantom perceptions [44]. The exact mechanism might be best evaluated by connectivity methodology in future studies; dynamic causal modelling would enable the modelling of the direction of information flow of assumed network hubs.

Our findings fit well to concepts and findings in tinnitus with respect to trans-synaptic chemical signalling. Inhibitory-excitatory dysbalance in tinnitus is considered to be related to altered activity of inhibitory-acting γ-aminobutyric acid (GABA) and excitatory-acting glutamate or *N-*methyl-d-aspartate (NMDA) [32]. Evidence comes from pharmacological treatments [32], animal models [45], and genetic analyses [46]. GABA and NMDA are also involved in motor cortex excitability and plasticity [31], [47]. Based on our findings it is tempting to speculate that the identified “trait-markers” *increased ICF* and *shortened CSP* reflect globally increased glutamatergic and reduced GABA-B transmission, respectively. Support for this theory comes from a recent genetic study providing preliminary evidence for a role of genetic variations of the GABA-B receptor in the pathophysiology of tinnitus [46].

In conclusion, present data show that tinnitus is accompanied by excitability and plasticity changes in the motor cortex. This finding sheds light on neglected areas in tinnitus research and highlights the inter-connectedness of the brain.
